# Novel imaging strategies for the detection of prosthetic heart valve obstruction and endocarditis

**DOI:** 10.1007/s12471-015-0796-0

**Published:** 2016-01-07

**Authors:** W. Tanis, R. P. J. Budde, I. A. C. van der Bilt, B. Delemarre, G. Hoohenkerk, J.-K. van Rooden, A. M. Scholtens, J. Habets, S. Chamuleau

**Affiliations:** 1 Department of Cardiology, Haga Teaching Hospital, Leyweg 275, 2545 CH The Hague, The Netherlands; 2 Department of Radiology, Erasmus Medical Center, Rotterdam, The Netherlands; 3 Department of Cardiothoracic Surgery, Haga Teaching Hospital, The Hague, The Netherlands; 4 Department of Radiology, Haga Teaching Hospital, The Hague, The Netherlands; 5 Department of Nuclear Medicine, University Medical Center Utrecht, Utrecht, The Netherlands

**Keywords:** Prosthetic heart valve, Endocarditis, Thrombosis, Pannus, Multidetector-row computed tomography, 18-fluorine-fluorodesoxyglucose positron emission tomography

## Abstract

Prosthetic heart valve (PHV) dysfunction remains difficult to recognise correctly by two-dimensional (2D) transthoracic and transoesophageal echocardiography (TTE/TEE). ECG-triggered multidetector-row computed tomography (MDCT), 18-fluorine-fluorodesoxyglucose positron emission tomography including low-dose CT (FDG-PET) and three-dimensional transoesophageal echocardiography (3D-TEE) may have additional value. This paper reviews the role of these novel imaging tools in the field of PHV obstruction and endocarditis.

For acquired PHV obstruction, MDCT is of additional value in mechanical PHVs to differentiate pannus from thrombus as well as to dynamically study leaflet motion and opening/closing angles. For biological PHV obstruction, additional imaging is not beneficial as it does not change patient management. When performed on top of 2D-TTE/TEE, MDCT has additional value for the detection of both vegetations and pseudoaneurysms/abscesses in PHV endocarditis. FDG-PET has no complementary value for the detection of vegetations; however, it appears more sensitive in the early detection of pseudoaneurysms/abscesses. Furthermore, FDG-PET enables the detection of metastatic and primary extra-cardiac infections. Evidence for the additional value of 3D-TEE is scarce.

As clinical implications are major, clinicians should have a low threshold to perform additional MDCT in acquired mechanical PHV obstruction. For suspected PHV endocarditis, both FDG-PET and MDCT have complementary value.

## Introduction

Valvular heart disease often requires prosthetic heart valve (PHV) replacement in order to improve quality of life and survival. The number of PHV implantations is expected to rise due to ageing, the growth of the population and the development of catheter-based techniques for valve replacement in patients who were inoperable before these techniques emerged. The major drawback of both mechanical and biological PHVs is development of dysfunction that is accompanied by high morbidity and mortality [1]. The reported incidence of PHV dysfunction varies from 0.01 to  6.0 % per patient-year and is dependent on valve position, valve composition, patient characteristics, patient compliance (endocarditis prophylaxis and anticoagulation in mechanical valves) and the nature of dysfunction. Causes of PHV dysfunction can be divided into three main groups that can be subsets of each other: (1) paravalvular leakage, (2) endocarditis and (3) obstruction [2].

As symptoms may be non-specific, echocardiography of PHVs plays a pivotal role and guides the therapeutic regimen. However, acoustic shadowing and reverberation of the metal of the PHVs hamper reliable imaging by transthoracic and transoesophageal echocardiography (TTE and TEE). For the detection of PHV leakages (not in the context of endocarditis) the combination of TTE and TEE has a high diagnostic accuracy for severity and location (valvular/paravalvular) and diagnostic dilemmas are mostly solved [3–6]. PHV endocarditis and obstruction often raise more diagnostic problems even after performing 2D-TTE/TEE [2]. Therefore the objective of this article is to review the additional value of new imaging modalities in the field of PHV endocarditis and obstruction. The diagnostic role of multidetector-row computed tomography (MDCT), 18-fluorine-fluorodesoxyglucose positron emission tomography including low dose CT (FDG-PET) and three-sdimensional TEE (3D-TEE) will be discussed. A diagnostic flowchart is provided that can aid in determining when and which modality to use in the clinical work-up of PHV endocarditis and PHV obstruction.

## PHV endocarditis

PHV endocarditis occurs with an incidence of 0.3–1.2 % per patient-year and is complicated by periannular extensions (pseudoaneurysms and abscesses) in at least 50 % of cases [7–9]. This is associated with an in-hospital mortality of 30 %, which may even rise to 50 % when *Staphylococcus aureus* is the causative bacterial agent [8–11]. The modified Duke criteria may have additional value in the establishment of the diagnosis of PHV endocarditis ([12]; Table 1). However, compared with native valve endocarditis, for suspected PHV endocarditis the two major Duke criteria are less reliable as the first major criterion (blood cultures) is often negative (23–37 %) and the second major criterion (echocardiography) is hampered by acoustic shadowing and reverberations [8, 10, 11].

**Table 1:** Modified Duke criteria for infective prosthetic heart valve endocarditis; in bold: the suggested change of the modified Duke criteria

***Major criteria***
*1. Blood cultures positive*
*Typical microorganism from two separate blood cultures:
Streptococcus viridans, Streptococcus bovis, HACEK Group, Staphylococcus aureus; community-acquired enterococci, in absence of a primary focus or
*Microorganism from persistently positive blood cultures:
At least two positive blood cultures drawn > 12 h apart or all of three or a majority of ≥ four separate cultures positive with the first and last drawn at least 1 h apart or
*Single positive blood culture for Coxiella burnetii or phase 1 IgG antibody titre > 1:800
*2. Imaging*
*Echocardiography: positive signs for infective endocarditis (vegetation, leaflet perforation, abscess, new partial dehiscence of the PHV)
*Molecular imaging (FDG−PET or leucocyte scan): for the detection of periannular infiltration
*Computed tomography: for the detection of periannular infiltration and/or vegetations
***Minor criteria***
*Predisposition: predisposing heart condition such as PHV or injection drug use
*Fever: temperature > 38 ℃
*Vascular phenomena: major arterial emboli, septic pulmonary infarcts, mycotic aneurysm, intracranial haemorrhages, Janeway lesions
*Immunological phenomena: glomeronephritis, Osler’s nodes, Roth’s spots, rheumatoid factor
*Microbiological evidence: positive blood culture that does not meet the major criterion
***Definite diagnosis of infective endocarditis***
2 major criteria
1 major and 3 minor criteria
5 minor criteria
***Possible diagnosis of infective endocarditis***
1 major and 1 minor criteria
3 minor criteria

The diagnostic value of TTE and TEE has recently been systematically reviewed and meta-analysed [2]. TEE was significantly more sensitive compared with TTE for the detection of vegetations (82 versus 29 %) and periannular extensions (86 versus 36 %) [2]. Although TEE detects the majority of signs of PHV endocarditis, the main finding of this study is that TEE misses the presence of vegetations (18 %) and periannular extensions (14 %) compared with the reference standard (i.e. surgical inspection/autopsy or clinical follow-up). Thus, ruling out PHV endocarditis by echocardiography incorporates uncertainty, clinically resulting in a low threshold to administer antibiotics for a prolonged period. On the other hand, periannular abscesses and pseudoaneurysms should not be missed as this is an indication for urgent surgical intervention.

## Novel imaging strategies for PHV endocarditis

### Multidetector-row computed tomography

**Fig. 1:**
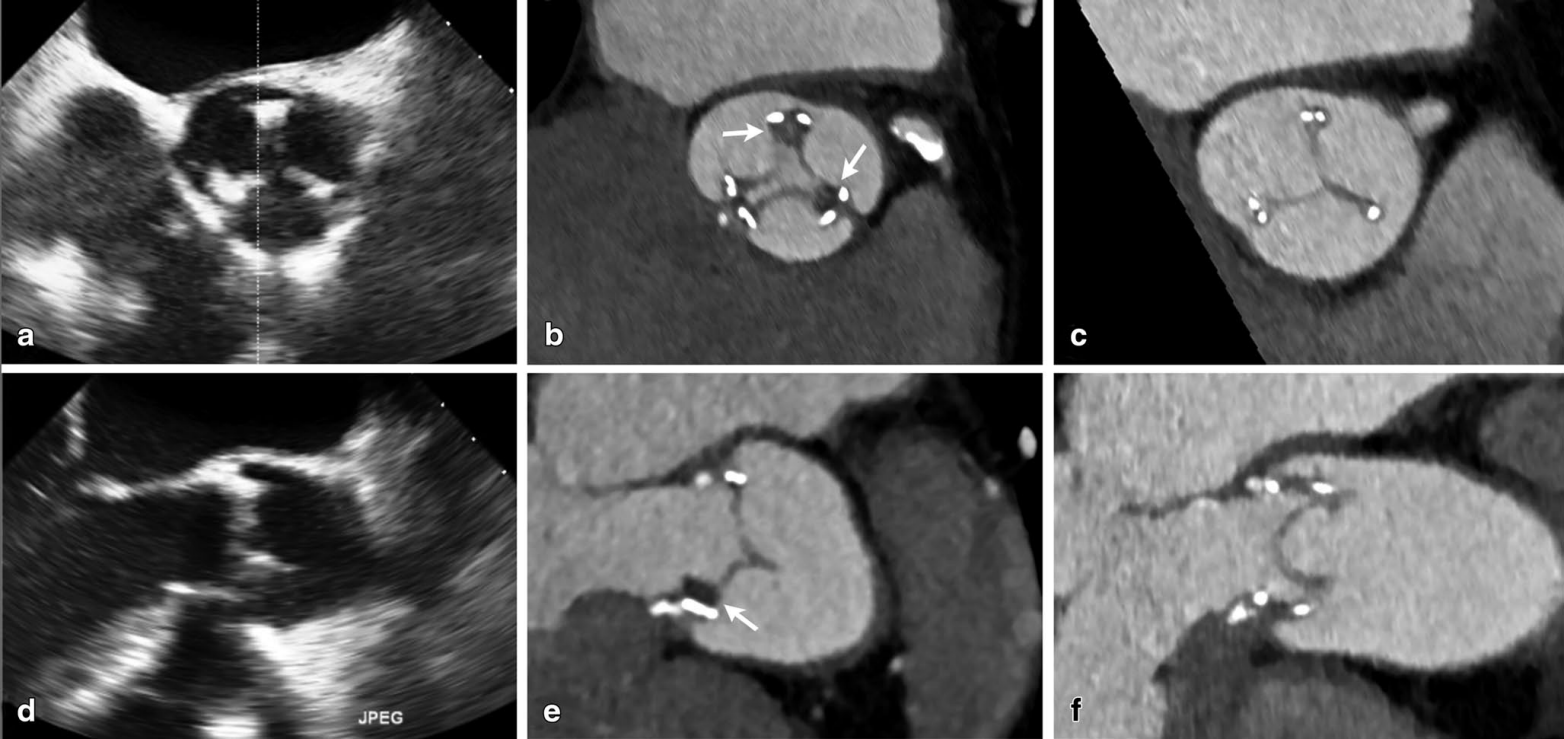
Vegetations missed by TEE. Patient with an aortic bioprosthesis for 3 years, presented with an infection of a knee replacement prosthesis (streptococcus). Blood cultures remained negative and TEE revealed no abnormalities (Panel **a** and **d**). CTA however detected hypodense masses (panel **b** and **e**, *arrows*) fitting with vegetations, which were successfully treated with 6 weeks of intravenous antibiotic treatment (panel **c** and **f**)

**Fig. 2:**
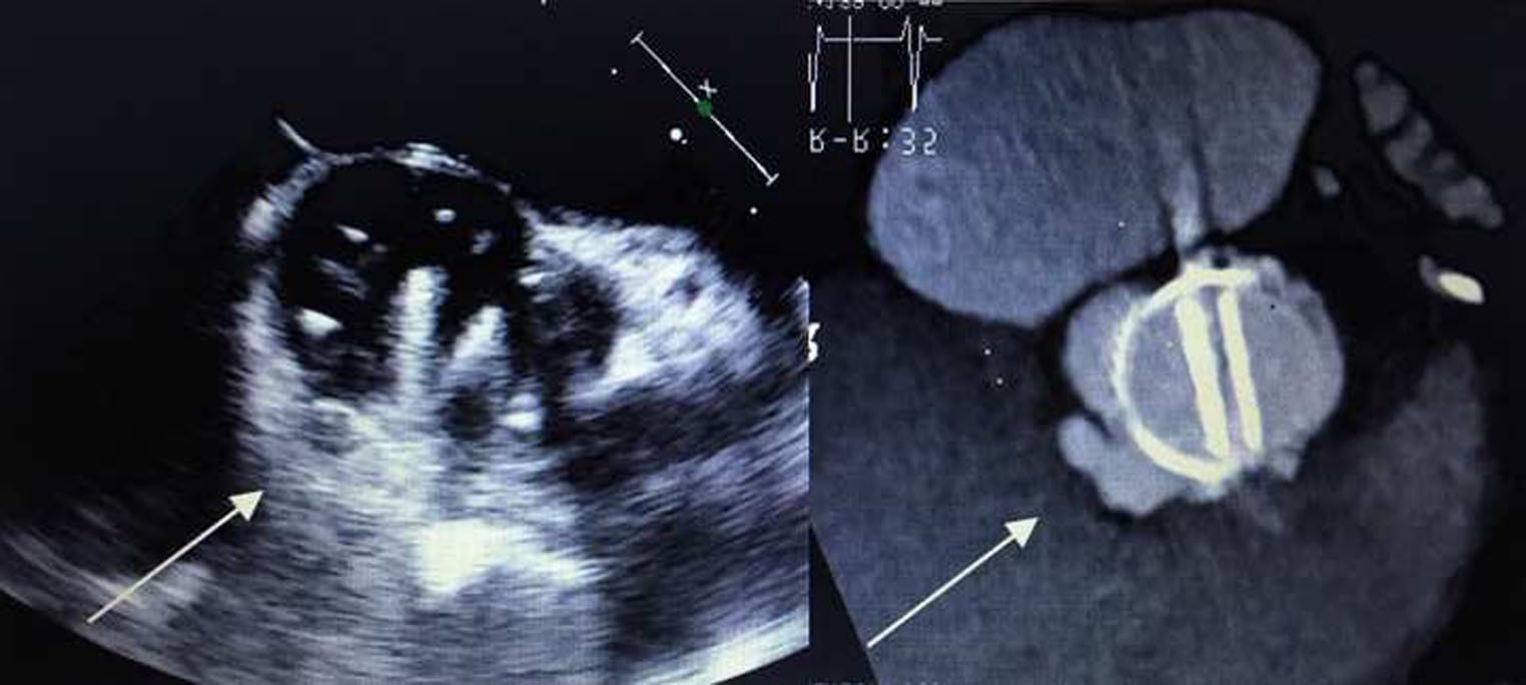
Pseudoaneurysm missed by TEE. Patient with an aortic mechanical prosthesis implanted 2 years before, presented with a Proprionii acnes bacteraemia. TEE missed the periannular extension (*arrows*)

MDCT has recently emerged as a promising novel imaging technique to evaluate PHVs. The technique uses electrocardiographic (ECG) gating to overcome artefacts related to the movement of the heart during the cardiac cycle. Reconstruction of the retrospectively ECG-gated acquired images provides 3D assessment of the PHV in any plane in each phase of the heart cycle. The acquisition is performed after the administration of contrast material. However, a non-contrast enhanced scan may have additional value to discriminate suture pledgets and calcifications of the PHV and can be performed using prospective ECG triggering in a single phase of the cardiac cycle. Contrast enhanced MDCT may detect PHV thrombi, pannus, vegetations, paravalvular leakages, mycotic aneurysms and even abscesses. In the field of PHV endocarditis three studies have been performed with MDCT [13–15]. These studies provide a strong indication that the diagnostic accuracy is improved when MDCT is added to TEE in the diagnostic workup (Fig. 1, 2). In one of the studies addition of MDCT resulted in a major diagnostic change in 6/28 patients (21 %), mainly driven by the novel detection of mycotic aneurysms [13]. Compared with the routine clinical work-up including TTE/TEE, addition of MDCT also resulted in a treatment change in seven patients (25 %). Therefore MDCT imaging has to be considered after clinical routine workup including TEE in patients with (suspected) PHV endocarditis.

### FDG-PET

**Fig. 3:**
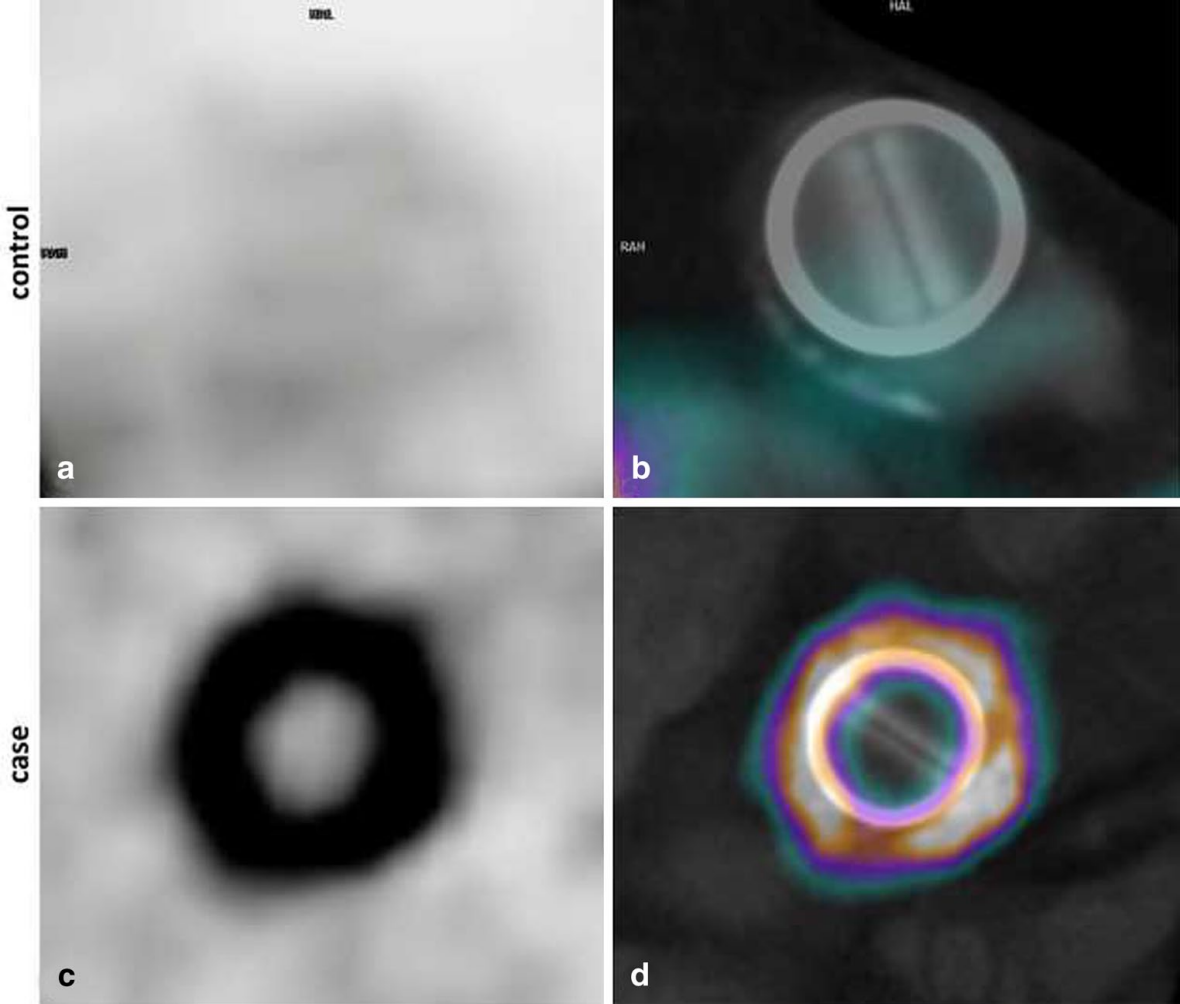
Case *versus* Control. Panel **a** and **b**: Aortic bileaflet mechanical PHV implanted 16 months previously, multiple blood cultures were positive for Proprionii bacterium. TTE and TEE did not show abnormalities and modified Duke criteria were not fulfilled. FDG-PET (**a**) showed severe FDG uptake. After fusion with MDCT, the FDG uptake was detected around the PHV ring (**b**), suggestive of widespread periannular extension of endocarditis around the whole PHV, which was confirmed at subsequent surgical inspection and pathological examination. Panel **c** and **d**: Aortic bileaflet St. Jude prosthesis in the chronic postoperative phase without suspected endocarditis. Metabolic imaging by FDG-PET on the level of the PHV did not show uptake. Re-printed with permission [20]

**Fig. 4:**
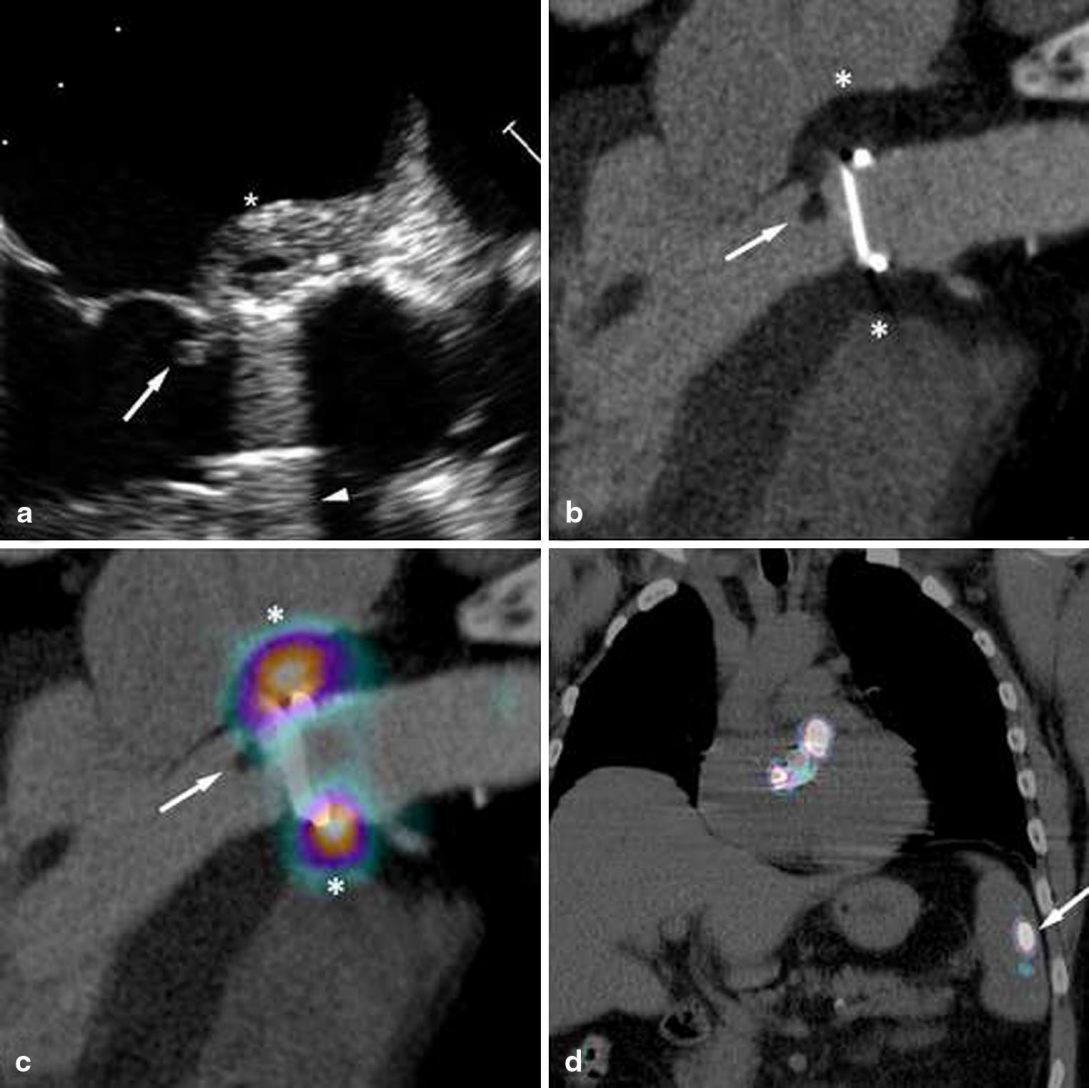
Metastatic infection in PHV endocarditis detected by whole body FDG-PET/low-dose CT. Bileaflet mechanical PHV in aortic position for 6 years, and seven consecutive blood cultures with Streptococcus pneumoniae. **a** TEE showed a vegetation (*arrow*) and thickened wall (*asterisk*) without colour Doppler flow in the former non-coronary cusp region, suggestive of an abscess. Former right coronary cusp (RCC) imaging is hampered by reverberations (arrowhead). **b** CTA confirmed TEE findings but also detected an abscess in the RCC region (*asterisks*). Panel **c**: CTA fused with FDG-PET confirmed abscesses (*asterisks*). The vegetation (*arrow*) did not show FDG uptake. Additionally, whole body FDG-PET/CT showed a metastatic infection in the spleen (Panel **d**, *arrow*), in this case an abscess requiring percutaneous drainage. Re-printed with permission [20]

FDG-PET is another imaging technique which is potentially useful in the diagnostic workup of these patients since it provides metabolic instead of anatomical information. PET scanning is based on the detection of annihilation photons released when the radionuclide 18 fluorine (18F) emits positrons. Unlike glucose, 18F-FDG is not metabolised but trapped in the human cell. Infected cells increase glucose consumption, resulting in accumulation of 18F-FDG [16]. However, cardiac myocytes have a relatively high glucose uptake compared with cells in other organs; therefore, it is important to force the heart to switch to fatty acid metabolism. This can be accomplished by a 24-hour low carbohydrate diet (last 12 h fasting). When heparin is infused (15 min before FDG administration) noninfected myocardial cells have even less FDG uptake [17]. As a result only infected cells take up 18F-FDG (Fig. 3). Recently, Saby et al. showed that the sensitivity and specificity of FDG-PET for PHV endocarditis was 73 and 80 % respectively [18]. However, when FDG-PET was added as a new major criterion to the modified Duke criteria (which includes TTE/TEE), the sensitivity rose from 70 to 97 %. The study also showed that FDG-PET alone missed a substantial number of vegetations (9/20, 45 %) in cases with no other echocardiographic signs of PHV endocarditis [18]. Most probably this is caused by motion of the valve leaflets and vegetations resulting in blurring of the PET signal beyond the point of detectability. Other contributing causes of missing vegetations may be the low spatial resolution of PET imaging, the background activity of the blood pool and/or direct exposure of vegetations to antibiotics in the bloodstream which make them more prone to be sterilised [19]. For this reason FDG-PET should always be combined with an imaging tool with better spatial resolution (TEE and/or MDCT) [20]. Combining MDCT and FDG-PET results in state-of-the-art high resolution anatomical and metabolic imaging of the PHV and its surrounding anatomy, which seems to be *the* desired imaging strategy in patients with high suspicion of PHV endocarditis but negative/inconclusive echocardiography ([20]; Fig. 4). Theoretically, the specificity of FDG-PET may be a concern as normal FDG uptake pattern in non-infected PHVs is poorly investigated. Especially in the early postoperative phase (< 1 year) healing and repair mechanisms may be a major issue and could result in false-positive imaging. Two small reports were published on the FDG uptake in non-infected PHVs [20, 21]. The results suggest that even in the early postoperative phase (from 6 weeks after PHV implantation) FDG-PET can be used as a diagnostic tool to rule out abscesses/pseudoaneurysms. Besides the detection of infection around PHVs, FDG-PET has an additional advantage as whole body PET is also able to detect extra-cardiac primary foci and/or metastatic infections (Fig. 4). In several studies metastatic/primary extra-cardiac infections were found in around 40 % of PHV endocarditis cases [22], resulting in major therapeutic consequences.

### Leucocyte scan

Single photon emission computed tomography (SPECT) is a 3D nuclear imaging technique. When using radioactively labelled leucocytes as the tracer it can be applied for the detection of infection. Images are obtained from multiple angles and are reconstructed in 3D. The study by Hyafil et al. investigated the additional value of detection of perivalvular infection in patients with a suspicion of PHV endocarditis and inconclusive TEE [23]. Diagnostic accuracy was not investigated; however in 12 out of 42 cases SPECT resulted in a change in patient management. Although labelled leucocyte SPECT/CT is much more time consuming and less sensitive compared with FDG-PET, SPECT is more specific especially in the early postoperative period. FDG-PET seems to be preferable as clinical implementation is easier and spatial resolution is superior. However, in the very early postoperative episode and in case of inconclusive FDG-PET, leucocyte SPECT scanning could be considered [23].

### Three-dimensional TEE

A major drawback of additional techniques such as MDCT and nuclear imaging is that they are expensive, expose patients to radiation and in case of MDCT iodinated contrast is administered. The use of contrast is relatively contraindicated when PHV endocarditis is complicated by renal failure. In most modern echo labs 3D-TEE is available for valve evaluation (anatomy) and guidance of treatment (e.g. TAVI, Mitraclip). This 3D technique may also be beneficial for detection of PHV endocarditis. 3D-TEE is performed with a multiplane probe including a 3D matrix-array. 3D full-volume datasets allow offline image editing using the multiple plane reconstruction mode and freehand cropping mode [24, 25]. This provides wide-angled 3D datasets with the ability to manipulate and crop images not limited to conventional 2D planar views. It enables visualisation of PHV valves with vegetations and periannular extensions, at angles not previously possible [25]. Two small studies investigated the complementary value of 3D-TEE. Both studies showed incremental value of 3D-TEE (on top of 2D TTE/TEE); however; compared with the reference standard, signs of PHV endocarditis were still missed and the number of included cases were very low.

### Cardiac magnetic resonance imaging

Cardiac MRI is safe beneath a value of 3 T for all modern PHVs (both biological and mechanical) as no significant interruption of valve motion nor overheating have been observed [26, 27]. The largest diagnostic problem is severe artifacts caused by the PHV resulting in a minimal additional imaging role of MRI for PHV endocarditis [28]. However, in the detection and quantification of PHV leakage, cardiac MRI may have a diagnostic role as phase contrast-based analysis can be subvalvular or supravalvular [29, 30].

### Proposed imaging strategy

**Fig. 5:**
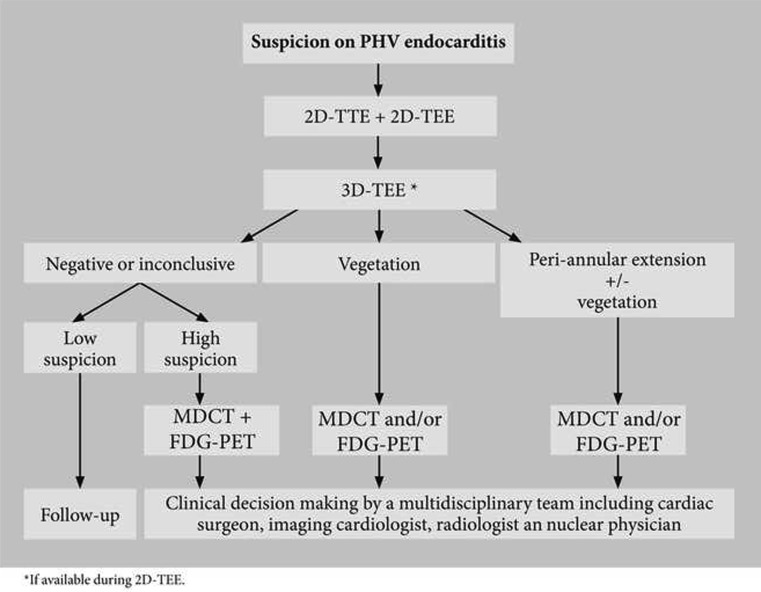
Imaging strategy for suspected PHV endocarditis

PHV patients with a suspicion of endocarditis should undergo TTE and TEE by an experienced sonographer. If 3D-TEE is available when performing 2D-TEE, one should have a low threshold to acquire 3D images of the PHV, at least for confirmation of the 2D findings. Patients with a high suspicion of PHV endocarditis and negative or inconclusive echocardiography should undergo additional MDCT and FDG-PET. In line with the recently published ESC guideline on endocarditis [31], we suggest to add MDCT and FDG-PET to the modified Duke criteria in case of PHV endocarditis suspicion (Table 1). In patients with already proven PHV endocarditis, addition of MDCT may still be favourable as it also supplies coronary angiography. From a surgical perspective it also provides information about the extent of periannular extension which guides the operation strategy. Addition of FDG-PET may still be useful when PHV endocarditis is already proven as extra-cardiac infections may be detected (primary/metastatic). Furthermore, when only PHV vegetations were detected by TEE, periannular extension may be ruled out or detected by additional FDG-PET and/or MDCT. Figure 5 shows a diagnostic algorithm regarding patients with suspected *and* proven PHV endocarditis.

## Concomitant coronary angiography

MDCT may replace preoperative invasive coronary angiography, which is not desirable in patients with aortic PHV vegetations as mechanical manipulation by an invasive catheter may be complicated by distal embolisation. Furthermore, in case of PHV reoperation MDCT provides valuable information about the distance between the sternum and the right ventricle and also the relation of the sternum to bypass grafts if present. In contrast to non-PHV cases [32], non-invasive angiography (MDCT) can be hampered by PHV scattering, especially in PHVs containing cobalt chrome (Bjork Shiley/Sorin tilting disk/Duromedics bileaflet) [33]. Moreover, MDCT protocols for PHV assessment use retrospective gating, whereas MDCT primarily performed for coronary assessment uses prospective gating and no beta-blockers or nitroglycerin [32, 34, 35] However, when conclusive, MDCT has a negative predictive value of 100 % compared with invasive coronary angiography (CAG) [36]. Following this strategy, in approximately 50 % of patients a preoperative CAG is no longer required, reducing radiation exposure, contrast use and complications. As could be expected, in patients with known coronary artery disease MDCT is not able to replace CAG.

### PHV obstruction

**Fig. 6:**
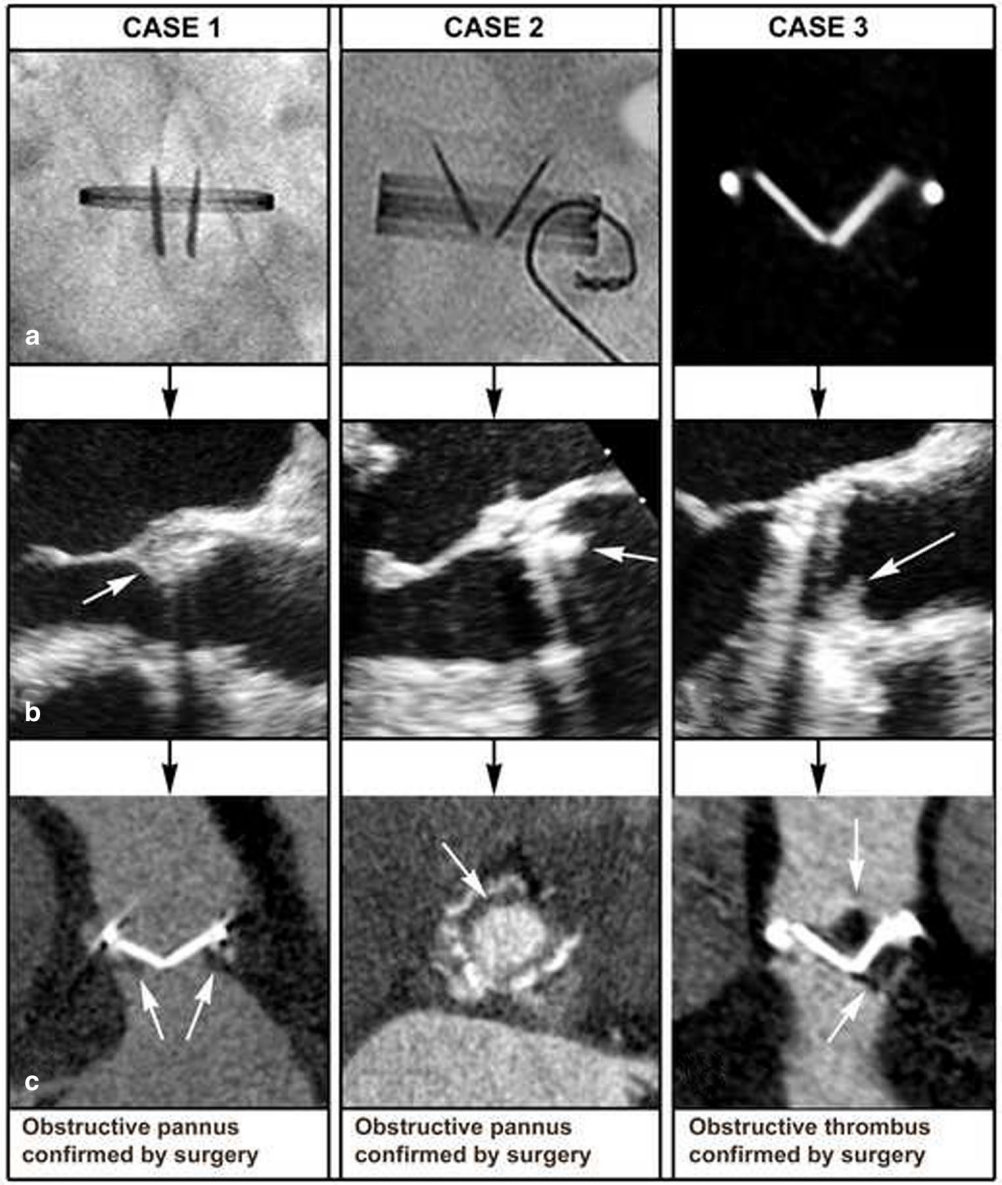
PHV obstruction case. Case 1: **a** Fluoroscopy; normal systolic opening angles of an aortic St. Jude mechanical PHV. **b** TEE; showing subprosthetic tissue (*arrow*). **c** MDCT; showing hypodense subvalvular tissue (*arrow*) curved along the PHV ring which was pannus confirmed by surgery. Case 2: **a** Fluoroscopy; both leaflets show systolic restriction. **b** TEE: aortic Tophat PHV its acoustic shadowing and reverberations (*arrow*). **c** MDCT shows hypodense subprosthetic tissue curved along the PHV ring which was pannus confirmed by surgery. Case 3: **a** Both leaflets show systolic restriction detected by MDCT. **b** TEE shows an oscillating mass at the aortic side of the St. Jude PHV. **c** MDCT shows an irregular shaped and hypodense mass directly attached to the occluder on the ventricular and aortic side which was thrombus confirmed by surgery. Re-printed with permission [43]

**Fig. 7:**
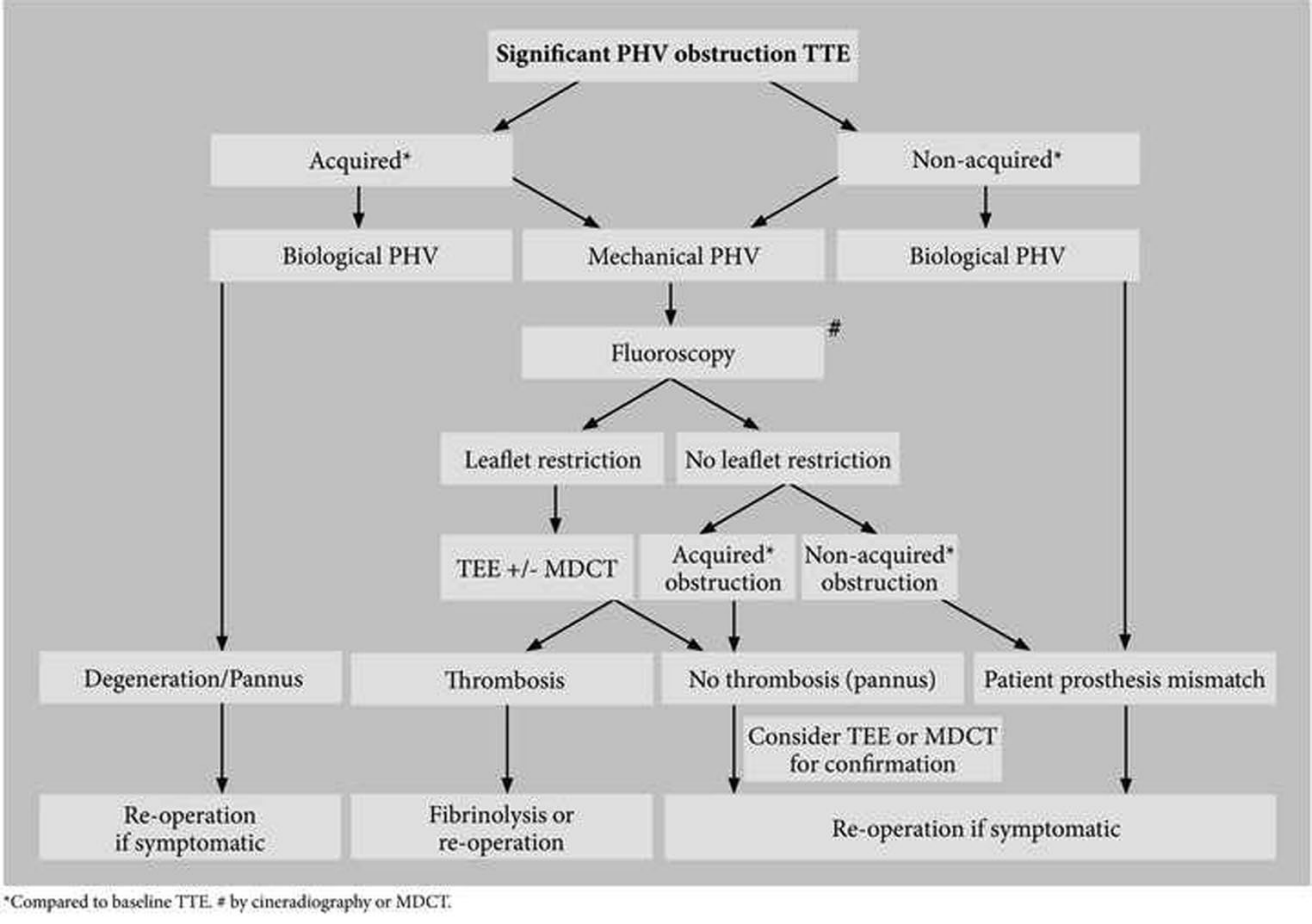
Imaging strategy for PHV obstruction

TTE is the first diagnostic screening tool with a good diagnostic accuracy for PHV obstruction. Transprosthetic valve gradients derived simultaneously by continuous wave Doppler and invasive catheter pressure measurements have a good correlation [37]. Biological and mechanical PHVs are susceptible to develop obstruction caused by either degeneration, pannus or thrombosis (acquired obstruction). In contrast to acquired PHV obstruction, patient prosthesis mismatch is present directly after the operation in adults. In the chronic postoperative phase, this mismatch can be differentiated from acquired obstruction by comparison of the effective orifice area of the initial postoperative outpatient TTE (around 6 weeks after implantation) and the most recent TTE [38]. This underscores the importance of this initial postoperative echocardiogram as advised by the ESC guidelines. In acquired obstruction the pathological mechanism between biological and mechanical PHVs is different. Acquired obstruction in biological PHVs is often calcifying degeneration, resulting in leaflet restriction. Theoretically another less frequent cause can be pannus formation around the stent struts or active endocarditis. The therapeutic consequence of significant obstruction of a biological PHV in a symptomatic patient is re-operation if clinically possible [38]. Therefore additional diagnostics are not required in biological PHV obstructions as they do not change patient management. In contrast to biological PHVs, acquired mechanical PHV obstruction is not caused by degeneration, but mainly by two entities: pannus or thrombus [39]. Differentiation between these causes is important as it may result in major therapeutic differences; obstructive thrombosis may require fibrinolysis and/or heparin infusion, whereas this is strictly contraindicated in obstructive pannus [39–41]. In clinical practice, the differentiation between obstructive thrombus and pannus remains challenging though very important when fibrinolysis is considered. Clinical parameters are not reliable enough to differentiate, therefore ESC guidelines advocate confirmation of thrombus formation by TTE, TEE and fluoroscopy [38]. Although the severity of obstruction can be reliably detected by TTE, it is not able to detect the exact cause of the obstruction [42]. A gradual decrease in effective orifice area suggests pannus; however, this information is often not present. Therefore the addition of fluoroscopy is advised. When the PHV is significantly obstructed and fluoroscopy does not show leaflet restriction, thrombolysis is not advised as pannus is confirmed (Table 2 and Fig. 6). Restriction of both leaflets detected by fluoroscopy is non-discriminative for pannus or thrombus [43]. Therefore ESC guidelines advise to add TEE in order to improve diagnostic accuracy. However, TEE assessment is hampered by acoustic shadowing caused by the PHV which contains metal and obscures adjacent anatomical structures for a correct diagnostic assessment. For this reason pannus and/or thrombus masses are often missed by 2D-TEE [42–47]. Only one dedicated study on the additional value of 3D-TEE in obstructed mechanical mitral PHVs was recently published [48]. This study shows that 3D-TEE has additional value on thrombus detection compared with 2D-TEE, but differentiation of thrombus from pannus was not investigated. Only a few studies have been published investigating the capability of 2D-TEE on this topic [43, 45, 46]. In case of mass detection thrombus/pannus differentiation by 2D-TEE remains difficult. Echocardiographic predictors for pannus/thrombus are shown in Table 2. However, differentiation may be provided more reliably by MDCT (Fig. 6). MDCT studies reveal that pannus is attached to the inflow site of the PHV ring and presents as a hypodense mass with a (semi)circular anatomical configuration curved along the valve ring. In contrast, obstructive thrombi are imaged as supravalvular and subvalvular hypodense masses with irregular anatomy directly attached to the leaflets and hinge points causing mechanical obstruction by leaflet restriction. Theoretically mass differentiation (thrombus versus pannus) may also be possible by determining the Hounsfield units, though no convincing evidence is available yet [43]. Furthermore MDCT enables visualisation of opening and closing kinetics of the PHV using a retrospective protocol with a minimum of 10 phases per heart cycle [49]. As outlined, MDCT is a promising imaging tool for correct differentiation of pannus from thrombus, however no prospective MDCT studies are present yet. Based on currently available literature the diagnostic algorithm is proposed as in Fig. 7.

**Table 2:** Fluoroscopy/Echocardiography/cardiac MDCT predictors for obstructive PHV pannus *versus* thrombus

**Pannus: fluoroscopy predictors**	**Thrombus: fluoroscopy predictors**
Leaflet restriction mostly present	Leaflet restriction always present
Leaflet restriction may be absent	Total occlusion of one leaflet and normal opening of the other leaflet (in bileaflet PHVs)
**Pannus: echo predictors**	**Thrombus: echo predictors**
Slow increase gradients/decrease EOA	Sudden increase gradient/decrease EOA
Absent/small mass on inflow side of PHV	Present mass/large mass on inflow + outflow side of PHV
Hard echo density	Soft echo density
**Pannus: MDCT predictors**	**Thrombus: MDCT predictors**
Hypodense mass on inflow side PHV	Hypodense mass on inflow and outflow side PHV
Mass anatomy: (semi)circular configuration curved along the valve ring	Mass anatomy: irregular shape, directly attached to the leaflets/hinge points

*EOA* effective orifice area, *MDCT* multidetector-row computed tomography, *PHV* prosthetic heart valve.

## General conclusion

PHV endocarditis and obstruction remain difficult to diagnose correctly by echocardiography alone. As clinical implications are major, this paper provides evidence that clinicians should have a low threshold to perform additional imaging by novel techniques in the field of PHV dysfunction, such as 3D-TEE, FDG-PET and/or MDCT.

### Disclosures

This work was supported by a grant from the Dutch Heart Foundation (NHS-2009B014)

Undisclosed authors did not contribute to this manuscript.
